# Use of Autotransplantation of Wisdom Tooth in Managing Oroantral Communication

**DOI:** 10.3390/jcm15176524

**Published:** 2026-08-24

**Authors:** Kinga Trepanowska, Michał Armatys, Bartosz Bielecki-Kowalski, Anna Dudko, Sebastian Kłosek

**Affiliations:** 1 Department of Periodontology and Oral Mucosal Disease, Medical University of Lodz, Pomorska 251, 92-213 Lodz, Poland; 2 Department of Oral Pathology, Medical University of Lodz, Pomorska 251, 92-213 Lodz, Poland; 3 Uno-Med Medical Center, 33-100 Tarnów, Poland; 4 Department of Maxillofacial Surgery, Medical University of Lodz, Zeromskiego 113, 90-549 Lodz, Poland

**Keywords:** oroantral communication, autotransplantation, third molars

## Abstract

Oroantral communication (OAC) is a known complication of maxillary tooth extraction that may lead to issues such as oroantral fistula formation and chronic maxillary sinus disease if left untreated. Although several surgical techniques for OAC closure have been described in the existing research, autotransplantation of a third molar represents a rarely reported alternative. This method not only allows for the closure of OAC but also restores masticatory function and may be an alternative to implantation. This review evaluates the available literature on third molar autotransplantation for OAC management, with particular attention to donor tooth selection, recipient-site preparation, surgical techniques, postoperative management, and clinical outcomes. The currently available literature on the mentioned topic is limited and consists predominantly of case reports and small clinical series. Nevertheless, published cases indicate that third molar autotransplantation may provide successful OAC closure and functional tooth replacement in appropriately selected cases. Significant heterogeneity and incomplete reporting of surgical and postoperative procedures, however, limit the strength of the available evidence. In this article, we propose a clinically oriented algorithm to facilitate decision-making in selected cases. This algorithm should be regarded as an expert-based framework requiring prospective clinical validation rather than an established standard of care.

## 1. Introduction

One of the risks regarding tooth extraction performed in the maxilla is the possibility of creating oroantral communication (OAC). OAC is an unnatural connection of the maxillary sinus with the oral cavity, most frequently occurring as a complication of extraction of the upper second molar [1]. The incidence of OAC varies depending on the study population, the type of procedure performed, and the anatomical relationship between the teeth and maxillary sinus; in large clinical studies, the incidence of OAC following surgical procedures in the posterior maxilla ranged from approximately 0.6% to 4.8%, although it may be significantly higher in patient groups with specific anatomical risk factors [2,3]. OACs present a slight preference to occur in male patients [4], and the causes include: anatomy (e.g., position of the roots, thickness of the bone between the tooth and the floor of the maxillary sinus), periapical inflammatory lesions, and iatrogenic actions [5]. Untreated OAC can lead to the creation of an oroantral fistula (OAF), which forms as a result of epithelialization of the OAC [4], and chronic antral infections with all of their consequences. There are also symptoms such as liquids passing from the oral cavity to the nostril on the same side as the OAC, unilateral nostril and intraoral discharge, foul taste and odor, and pain in the same-side cheek [4]. OACs’ severity depends on the size of the orifice in the alveola.

Third molar extraction is one of the most frequently performed oral surgical procedures [6,7] and can be associated with various complications; the incidence of these depends on factors such as tooth location and the need for more invasive surgical techniques. Therefore, when planning the autotransplantation of a third molar, the assessment of the potential donor tooth should consider not only its morphology and local condition but also the feasibility of atraumatic extraction, as preserving the integrity and viability of periodontal ligament cells is crucial for the procedure’s success.

Although third molar autotransplantation for OAC closure has previously been described in individual case reports and small clinical series, the available evidence remains deficient, as this treatment option has received minimal attention in broader reviews of OAC management. To the best of our knowledge, no previous review has specifically synthesized the existing literature concerning closing OAC using third molar autotransplantation while integrating patient and donor tooth selection, recipient-site preparation, surgical techniques, postoperative management and follow-up into a clinically oriented framework. Therefore, the originality of this review lies not in introducing the surgical technique itself, but rather in its focused synthesis and critical appraisal of the available evidence, as well as in the development of a proposed clinical algorithm intended to support decision-making and identify areas requiring further clinical validation.

The aim of this study is to analyze cases and present the advantages and disadvantages of closing oroantral communication using the autotransplantation of the third molar. The paper focuses on a review of the existing literature regarding the previously mentioned procedure, comparing it with other methods of post-extraction treatment of the connection between the oral cavity and the maxillary sinus.

## 2. OAC—Risk Factors and Possible Treatment Options

Although OAC most frequently arises as a result of the extraction of maxillary molars, it can also occur after the extraction of upper premolars, particularly second ones [2]. In such cases, the use of autotransplantation requires consideration not only of the possibility of achieving a tight closure of the communication and a proper fit of the donor tooth to the recipient site, but also of the aesthetic outcome, which gains greater importance in the more anterior region of the dental arch.

There are few methods of diagnosing an OAC. Firstly, an intraoral examination needs to be conducted. It allows assessing the surgical field and spotting OACs of a bigger size. Valsalva test (closing the patient’s nostrils while the patient is exhaling through them), in OAC cases, results in bubbling of the blood in the alveoli and creates a whistling sound. It should be noted here that the Valsalva maneuver can often yield a false-negative result in the presence of concurrent changes in the maxillary sinus. Furthermore, if performed improperly (with excessive pressure), it can lead to laceration of the sinus mucosa by the sharp edges of the alveolar ridge [2]. We can also carefully explore the communication with the periodontal probe [4]. Radiology helps with the diagnosis as well. Both 2-D (periapical, occipitomental, and panoramic) and 3-D imaging (CT or CBCT) can be used. 2-dimensional radiographs allow assessment of the anatomy of the maxillary sinus and alveolar ridge, and diagnose any foreign objects within these anatomical structures. CT or CBCT are more precise and can also determine soft tissue status [1].

Cases with openings of size 5 mm or less often do not require intervention as they can heal spontaneously [8]. More severe clinical situations, i.e., larger perforations or those that last more than 3 weeks [9], necessitate surgical treatment. OACs can be managed in many ways, including the Wassmund–Borusiewicz method, Zange method, buccal advancement flap, buccal musculocutaneous flap, palatal arterialized flap, pontine flap, lingual flap, usage of Bichat fat pad, and double-layer closure [10], of which the most common is the buccal advancement flap [11]. There are numerous alternatives: variations of buccal flaps such as the Môczáir flap [12], use of alloplastic materials, and nonporous hydroxyapatite [13]. One of the methods used for immediate closure of OAC is the use of PRF (platelet-rich fibrin) [14]. Salgardo-Peralvo et al. [15] report that PRF can be used to close sinus connections up to 5 mm in size—in the case of larger perforations, the authors recommend combining this technique with other bi- or triluminal procedures. This method has relatively few side effects and is characterized by minimal postoperative morbidity compared to the surgical procedures mentioned above. This article aims to examine a less widely recognized approach to this problem. In cases where there are indications to both remove the upper wisdom tooth and post-extraction OAC presented, the third molar can be used for the closure of the communication [9].

## 3. Autotransplantation—Procedure Description

Tooth autotransplantation is the replacement of a missing permanent tooth with another autogenous impacted or functionally irrelevant tooth [16]. Autotransplantation should be distinguished from replantation, which involves placing a previously extracted tooth back into its original socket [16] and may be considered, for instance, in cases of accidental tooth extraction [17]. Autotransplantation finds application also in other clinical situations—for example in cases of anterior tooth loss—where aesthetic outcomes are of particular importance alongside biological and functional success; however, these issues fall outside the scope of the present review, which focuses on the use of third molars in OAC management.

The principles of anesthesia for the procedure do not differ from those used for the removal of impacted teeth, and the same instruments are used in both cases. When removing a tooth intended for transplantation, it is important to ensure that it is not damaged: maintaining the viability of periodontal cells is crucial; hence, the extraction should be minimally traumatic [18]. In cases of fully erupted teeth, dental forceps as well as elevators are the most commonly used instruments. When dealing with impacted teeth, a gentle osteotomy is performed so that the dental sac is not damaged during the procedure [19]. It is also very important that the time between extraction and replanting the donor tooth is minimal [9] because it determines the number of living periodontal cells, which are a key factor in determining the success of this treatment. The literature includes cases of immediate as well as delayed autotransplantation, since the operator responsible for the treatment is not always able to perform the procedure right after detecting the OAC.

Assad et al. [20] as well as Nagori et al. [21] mention placing a saline-soaked gauze in the recipient site after extraction.

The recipient socket should resemble the one after extraction, and for this purpose it can be modified using round surgical drills—for instance, round surgical bur no. 8 [11]—at a low speed while being cooled with saline solution [13]. Yu et al. reported using Thommen Medical AD dental implant drills for this procedure [18]. Since the described cases involve closing the connection after extraction of the upper molars, the modification of the recipient site most often involved the removal of the [21] septum in cases where the replanted tooth was single-rooted. Currently, the standard procedure in the case of tooth autotransplantation is shaping the recipient site based on a 3D-printed model of the donor tooth [22,23,24]. The model is designed based on pre-extraction CBCT. Hou et al. [23] report using a PLA (linear polylactic acid) model, as this material provides good stability, toughness, and durability as well as great water resistance. This material is also non-toxic. After placing the tooth in the recipient socket and verifying its stability, it should be immobilized with proper sutures: preferably mattress stitches crossed over the occlusal surface [25]. Other methods include splinting and using manual pressure as well as light tapping.

Usually, the teeth used for closing the OAC have completed root development [18].

When we replant a tooth that is mature, i.e., its apical foramen is closed, the probability of maintaining pulp vitality is very low [25]: only 15% versus a 96% rate as is in the case with immature teeth [26]. Therefore, it is recommended to perform endodontic treatment: the American Association of Endodontics recommends performing pulp extirpation within 7 to 14 days after transplantation [18] in order to minimize the risk of developing infection from necrotic pulp. RCT could also be performed after the donor tooth has been extracted, but before placing in the recipient site—although this approach is not advised due to prolonged extraoral time, which can affect the longevity of periodontal cells and thus increase the risk of root resorption development. In case of immature tooth autotransplantation, a pulp vitality test is advised. Such checkups are usually performed about 6 weeks post-procedure [18]. If the reaction to the electrometric pulp test is negative, endodontic treatment should be carried out as well. Pulp vitality assessment of the transplanted tooth should include multiple testing modalities, as no single method provides complete diagnostic accuracy. Conventional sensibility tests, including thermal (cold and heat) testing and electric pulp testing (EPT), evaluate pulp status indirectly by measuring neural response rather than actual blood supply. Current pulp sensibility testing methods do not take into account vascular circulation, resulting in false-positive and false-negative results, particularly in teeth that have temporarily or permanently lost their sensory function [27]. This limitation is especially relevant following autotransplantation, where reinnervation significantly lags behind revascularization. Laser Doppler flowmetry (LDF) and pulse oximetry achieve higher diagnostic accuracy compared to thermal and electrical pulp testing, with heat testing being the least accurate among all available methods [27,28]. LDF, by directly measuring pulpal blood flow, provides the most objective and reliable assessment of pulp vitality, and its use should be considered in the post-transplantation follow-up protocol wherever available [28,29].

## 4. Post-Procedure Guidelines

**Figure 1:**
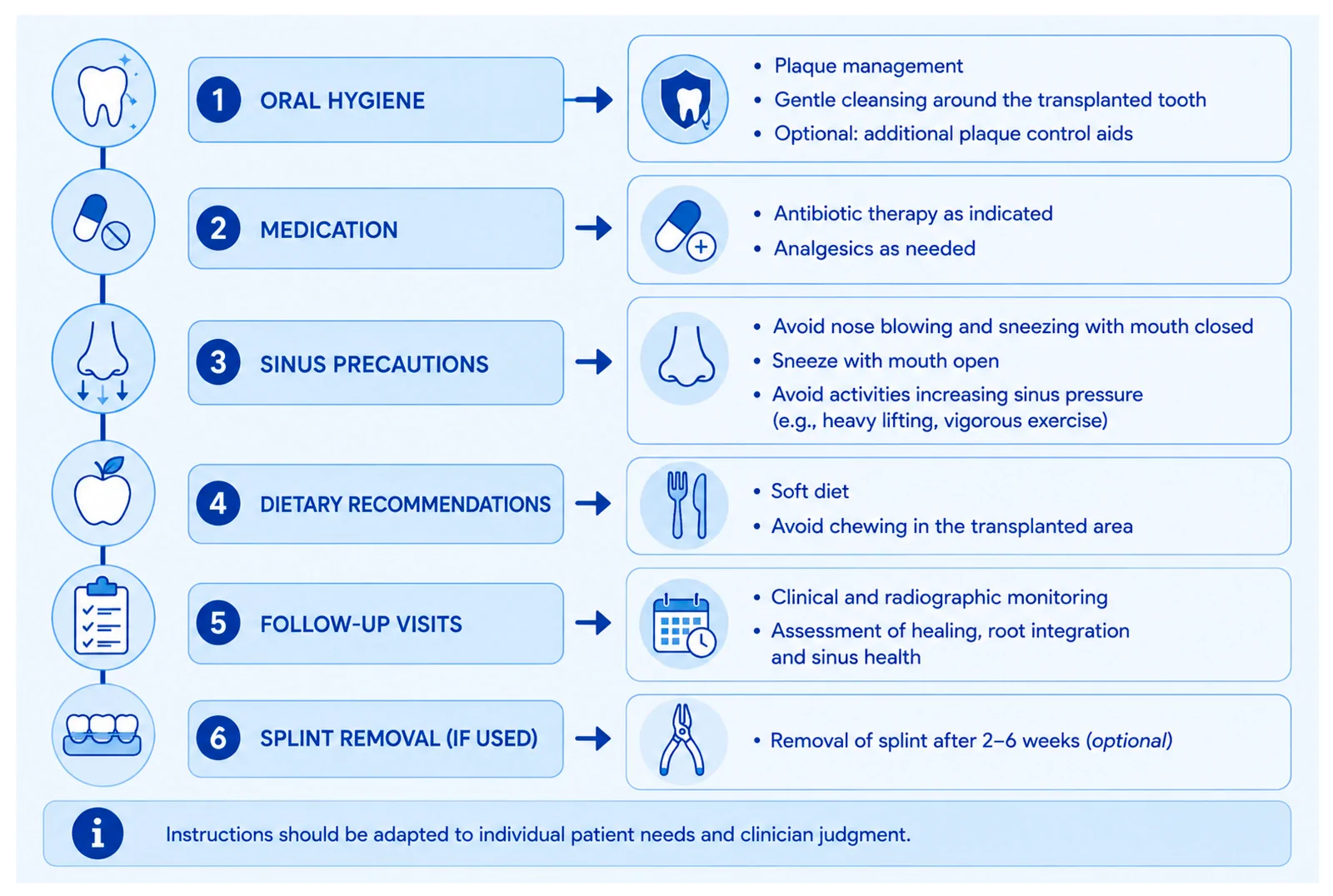
Overview of the proposed postoperative care following third molar autotransplantation for OAC closure.

Available reports suggest the use of chlorhexidine rinse and antibiotic therapy lasting from 3 to 7 days. [11,14]. Assad et al. mention amoxicillin and clavulanic acid administered three times per day [21]. In some cases, anti-inflammatory drugs are also recommended [11]. Indications detailed in the Recommendations of the Working Group of the Polish Dental Association and the National Antibiotic Protection Program (PTS/NPOA—Polskie Towarzystwo Stomatologiczne/Narodowy Program Ochrony Antybiotyków) regarding the use of antibiotics in dentistry state that surgical-site infection prophylactic administration of a single dose antibiotic is recommended for procedures involving opening the maxillary sinus lumen. Those involve a single dose of 2000 mg amoxicillin p.o. (in case of allergy to penicillin antibiotics: 1000 mg cefazolin or 600 mg clindamycin) 30–60 min prior to the procedure [30]. In the case of children, 50 mg/kg b.w. amoxicillin or 20 mg/kg body weight clindamycin is administered. This approach is known as ‘one-shot’ antibiotic prophylaxis. These recommendations are consistent with the European Society of Endodontology position statement on the use of antibiotics in endodontics [31] and the ESE S3-level clinical practice guideline for the treatment of pulpal and apical disease [32], both of which advocate for targeted, short-course antibiotic therapy and prophylactic protocols aligned with antimicrobial stewardship principles [33]. It should be emphasized that the above recommendation applies solely to the prevention of surgical site infection (SSI) and not to the prophylaxis of infective endocarditis (IE); these two indications should not be equated as they are characterized by different clinical criteria. Furthermore, the PTS/NPOA guidelines do not recommend routine antibiotic prophylaxis for every patient undergoing tooth autotransplantation—the single-dose regimen described above applies specifically to procedures involving opening of the maxillary sinus lumen or the removal of extensive maxillary bone lesions, whereas prophylaxis is not routinely indicated for immunocompetent patients undergoing other endodontic surgical procedures. In the PTS/NPOA recommendations, clindamycin is listed as an alternative for SSI prophylaxis in patients with penicillin allergy. However, this recommendation must be interpreted within the context of the specific indication and should not be directly extrapolated to other indications for the prophylactic or therapeutic use of antibiotics. This must be distinguished from the regimens proposed for IE prophylaxis, where clindamycin is no longer recommended under the 2023 ESC guidelines for the management of infective endocarditis due to an increased risk of severe adverse effects, including C. difficile infection [34]. According to the 2023 ESC guidelines, for patients at the highest risk of IE undergoing dental procedures involving manipulation of gingival tissue, the periapical region of the tooth of perforation of the oral mucosa, the first-choice antibiotic remains a single dose of amoxycillin or ampicillin (2000 mg for adults, 50 mg/kg body weight for children) administered 30–60 min prior to the procedure; whereas for patients allergic to penicillins, recommended alternatives include cephalexin, azithromycin or clarithromycin, and doxycycline, or—for parenteral administration—cefazolin or ceftriaxone (however, cephalosphorins should not be administered to patients with a history of anaphylaxis, angioedema or urticaria following penicillin/ampicillin administration). Nevertheless, given the growing amount of data regarding adverse effects associated with clindamycin use, some authors advocate for a more restrictive approach to its use—including in the prophylaxis of IE—and the risk-benefit balance should be assessed on an individual basis by the attending physician in each case. Given the differences in indications for antibiotic use in the perioperative and postoperative periods, the key recommendations regarding SSI prophylaxis, IE prophylaxis, and therapeutic antibiotic therapy are summarized in Table 1. Overview of the proposed postoperative care is presented in Figure 1 below.

## 5. Contraindications

Despite its wide use and apparent ease of the procedure, it is not always possible to undertake it in practice. Apart from contraindications, which make the procedure impossible or unsafe to perform, risk factors are also distinguished. Contraindications can be divided into two groups: absolute and relative.

Absolute local contraindications to the procedure include insufficient space for the donor tooth, presence of inflammatory root resorption and pathological lesions, as well as damage to the periodontal and periosteal tissues [9]. Local changes in hard or soft tissues such as sinus infection, acute infections of dental origin, previously mentioned pathologies such as polyps, cysts, and tumors, as well as sinusitis, allergic rhinitis [35]—all require healing before autotransplantation surgery. Each of the above-mentioned ailments may affect the course of treatment.

This procedure cannot be conducted in large OACs or in cases of OAFs (oroantral fistulas) [21].

The patient’s risk factors include [36]: the presence of cardiovascular diseases and taking anticoagulants—both factors increase the risk of excessive bleeding during the procedure; however, the use of anticoagulants alone does not constitute a contraindication for most dental procedures and should not habitually lead to the arbitrary discontinuation of therapy [37]. Risk must be assessed on an individual basis, taking into account the type of medication, the extent of the procedure, and the feasibility of achieving local hemostasis; diabetes—uncontrolled hyperglycemia is associated with increased susceptibility to infections due to impaired immunity, studies have shown that diabetes in the patient is associated with post-operative swelling, bleeding, flap dehiscence, and delayed wound healing; osteoporosis and taking antiresorptive drugs such as bisphosphonates—their supply increases the risk of MRONJ (medication-related osteonecrosis of the jaw). Relative contraindications to the procedure also include patients’ alcohol abuse, drug addiction, or the presence of mental disorders, as they may affect patient cooperation (i.e., post-operative oral hygiene) and the healing process [35]. In the event of sepsis or other severe systemic diseases, the procedure is also waived. Radiotherapy to the maxillofacial region and the use of antiresorptive agents should not be regarded as absolute contraindications; rather, they are recognized risk factors requiring thorough pre-procedural workup and targeted prophylaxis [38].

Relative contraindications that make it difficult to perform the procedure and maintain its effects include limitations in opening the mouth; malocclusion, and severe bruxism [35]. The global prevalence of bruxism has been estimated at 22.22%—as a factor that may increase the load on the stomatognathic system, it should be taken into account during patient screening and the assessment of potential treatment-related risk factors [39].

## 6. Literature Review

There are few studies in the literature regarding the closure of the OAC with third molars. Assad et al. [20] studied 20 patients aged 20 to 40 who underwent the procedure. In the case of this study group, the procedures were planned—after detecting OAC resulting from upper 1st or 2nd molar extraction, patients were referred to the maxillofacial surgery clinic. The vast majority of OACs resulted from 1st upper molar extraction. After being extracted, the donor tooth was kept in a solution of saline and penicillin; a solution of the same composition was used to rinse the sinus fistula. Each third molar used in the mentioned study underwent endodontic treatment after extraction and before placement in the recipient site. After a one-year follow-up examination, the effectiveness in closing the OAC reached 95%. In 90% of cases, tooth autotransplantation was successful [21]—this indicates that not in every case in which the connection was closed, the tooth was kept in the recipient socket.

Another clinical report provided by Kitagawa et al. concerns two female patients, one of whom presented with left sinusitis caused by a periapical granuloma [9]. Drainage through a mucosal incision and antibiotic therapy (in this case, the author does not reveal which drug was administered) were used in order to alleviate the symptoms. One month later, CT showed resolution of inflammation in the sinus. After the removal of the causative tooth, which was the first upper molar, scraping of periapical lesions resulted in disruption of the sinus mucosa. Autotransplantation of the third molar was performed immediately after identifying the OAC and removing the septum in the recipient site (the donor tooth had one conical root). The second examined patient was qualified for the procedure right after it was found that there was an insufficient amount of maxillary alveolar bone for potential implantation after the extraction of the first upper molar. Instead of using a bone graft, the lower third molar was replanted in place of the extracted tooth immediately after removal. Both procedures were uneventful, as confirmed with post-operative examinations after a couple of months: in the case of the first patient, the sinusitis symptoms disappeared completely; both cases healed without any signs of ankylosis or root resorption; furthermore, both of the donor teeth showed good stability in recipient sockets [9]. Nagori et al. [21] reported a similar case in a 22-year-old female patient who was scheduled for extraction of the first upper molar with extensive destruction of the crown. After the procedure, the socket was curetted to remove the periapical lesion, which resulted in the formation of an OAC. Based on the pre-operative OPG, it was decided to immediately replant the left upper wisdom tooth, which in this case was impacted. The choice of the donor tooth was motivated by the fact that it was easier to obtain than the right counterpart. After removing the septum from the recipient site (the replanted tooth had a single root) and securing it with a gauze soaked in saline solution, the third molar was atraumatically removed and immediately placed in the socket of the first molar, then stabilized with sutures and additionally splinted with a stainless-steel wire. Post-operative pharmacotherapy included the patient taking amoxicillin three times a day, as well as an antihistamine and analgesic. A semi-liquid diet was recommended until RCT. The sutures were removed after a week, the splinting after 3 weeks, at which time endodontic treatment of the transplanted tooth was also performed. After 1.5 years, a follow-up examination showed the presence of lamina dura as well as the periodontal ligament, and the patient was asymptomatic. Another paper by Grün et al. [19] describes the case of a 20-year-old patient who presented with a radicular cyst originating from the previously endodontically treated tooth 26. The chosen treatment method was apical resection and removal of the cyst, and six months after the procedure, tooth 26 was extracted due to its fracture. This resulted in the formation of OAC. Potential implantation would require augmentation, and extensive damage to the sinus mucosa significantly hampered further clinical management, so it was decided, in consultation with the patient, to replant the impacted tooth 28 in place of the removed first molar. This procedure didn’t require RCT of the replanted tooth because of its incomplete root development. The donor tooth remained in the oral cavity for as long as 19 years. Unfortunately, progressive external resorption resulted in its final extraction. Subsequent implantation did not require augmentation due to the healing of the mucous membrane and the development of bone in the autotransplantation area.

To evaluate the success of the procedure, Assad et al. divided the criteria into radiographic and clinical. Radiographic criteria include:

- Normal width of the periodontal space around the autotransplanted tooth.
- No signs of root resorption.
- Corticalisation (radio-opaque line at the septal bone/lamina dura).
- Preserved sinus patency—no changes or reduction of pre-existing changes.
- Reconstruction of the sinus floor contour with bone formation.

Clinical criteria consist of:

- No signs of pathological tooth mobility (checked with Periotest).
- No signs of inflammation.
- No signs of OAC.

Presence of an inflammatory process, root absorption or root fracture, as well as evidence of periodontal attachment loss, suggests a failed procedure [20].

The procedure described in this paper has many undoubted advantages but also its drawbacks and possible complications that may be responsible for treatment failure.

Risks of failure concerning the procedure include insufficient bone amount surrounding the recipient site; age (autotransplantation of teeth with incomplete root development has a higher rate of success); presence of systemic diseases as well as inflammatory lesions and ongoing oncological treatment and/or immunosuppressive treatment; complicated anatomy of the third molar region. The main advantages and disadvantages of the presented method are summarized in Table 2. The characteristics, treatment protocols and outcomes of the studies included in this review are presented in Table 3.

## 7. Proposed Clinical Algorithm for OAC Closure Using Third Molar Autotransplantation

**Figure 2:**
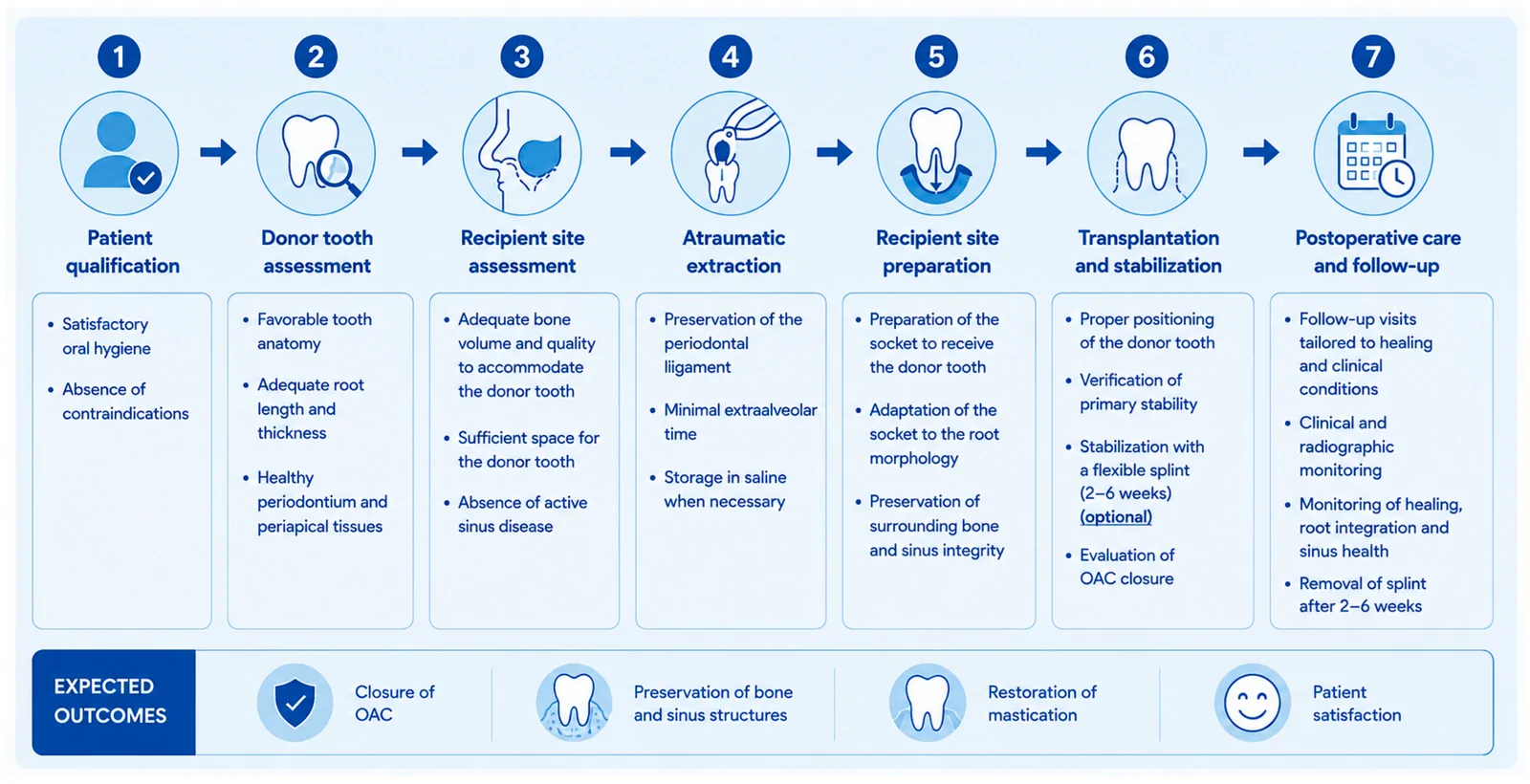
Clinical protocol for closing an OAC using third molar transplantation.

Due to the limited number and methodological quality of currently available studies, the following algorithm should not be regarded as an evidence-based clinical guideline or an established standard of care. It constitutes a proposed clinical algorithm developed through a synthesis of existing literature on the autotransplantation of third molars to close oroantral communications (OAC). Its aim is to provide a structured framework for clinical decision-making in this uncommon scenario. Further clinical research is required to validate the proposed method and to determine its safety, efficacy, and reproducibility. The proposed clinical algorithm is presented in Figure 2.

### 7.1. Patient Qualification

- Older age is related to impaired healing processes, a higher infection rate due to increased caries and periodontal disease risk, and more difficult extractions linked to increased bone density [11].
- Systemic diseases, nutritional deficiencies, as well as hygiene level may influence and disturb the healing process, in some cases making it impossible to perform this treatment.

Fundamental diagnostic tools in this case include a thorough medical anamnesis paired with the results of the patient’s blood tests for indicators of inflammation that may be occurring in the body, as well as nutritional deficiencies.

If the considered patient meets the qualification requirements for the procedure, we subsequently analyze the potential donor tooth condition.

### 7.2. Donor Tooth Condition

- Absence of pathologies such as periapical lesions, root resorption, and caries.
- Anatomy that enables atraumatic extraction followed by placing an undamaged, whole tooth in the recipient site is simpler in terms of both extraction and autotransplantation, as its straightforward root morphology facilitates atraumatic removal and reduces the risk of root fracture during the procedure.

Assuming that the tooth we aim to use for OAC closure has favorable anatomy and isn’t damaged, nor are inflammatory lesions present, we then proceed to recipient site qualification. CBCT is considered the most detail-giving radiological diagnostic tool here—this particular form of radiological diagnostics plays a crucial role in planning autotransplantation, enabling a three-dimensional assessment of the donor tooth’s morphology and the anatomical conditions of the recipient site. CBCT data can also be used to create a 3D replica of the donor tooth, allowing for recipient site preparation prior to extraction and minimizing both the extra-alveolar time and the number of trial fittings for the transplanted tooth [20].

### 7.3. Recipient Site Qualification

There are several factors influencing the procedure’s success rate:

- Space for the donor tooth between adjacent teeth.
- Size of the OAC.
- Presence of inflammatory lesions such as cysts in the maxillary sinus.

If there is enough space, the OAC is small enough, and there are no signs of any infection going on in the sinus, then the procedure can be performed. Based on the latest clinical data provided by Bumairemu et al. [40], a size range of 5–8 mm can be identified as the documented range for OACs suitable for immediate autotransplantation; however, given the limited number of available studies, this range should not be regarded as a validated eligibility threshold.

### 7.4. Recipient Site Preparation

The recipient alveolar socket may need preparation in order for the donor tooth to fit. Considering the crucial role of periodontal cells in successful transplantation, the donor tooth should not be outside of a socket for more than is necessary to replant it to the recipient socket, nor should it be moved around in and out of a recipient socket as a guide to prepare the socket’s shape. Preparation of the recipient site should be as conservative as possible, maximizing the preservation of existing bone tissue and limiting preparation to the extent necessary to achieve a proper fit and stability for the donor tooth. When using a digital model or a 3D replica of the donor tooth, it is possible to prepare the recipient site more precisely prior to extracting the donor tooth. This approach can reduce the number of fitting attempts, shorten the extra-alveolar time, and lower the risk of mechanical damage to the periodontal ligament. A systematic review by Verweij et al. [41] indicates that the use of a 3D replica of the donor tooth enables precise planning of tooth position and reduces extra-alveolar time, although the quality of available evidence regarding this technique remains limited. To avoid damage to periodontal cells, alveolar bone can be gently shaped with a low-speed handpiece and surgical bur/implant bur to resemble a donor tooth bed; however, the operator should bear in mind that excessive widening of the socket can lead to unnecessary bone loss and compromise periodontal healing conditions. Consequently, the use of large-diameter implant drills should not be considered a routine step in the mentioned procedure. After recipient site preparation, there should be enough space for the loose fitting of the donor tooth [18].

Regarding the problem of insufficient bone volume at the recipient site, the feasibility of autotransplantation should be assessed on an individual basis. In cases of significant bone deficiency that prevents achieving proper positioning or stability of the donor tooth, regenerative procedures—including guided bone regeneration (GBR)—may be considered in conjunction with autotransplantation. Available reports indicate that such an approach may be effective in selected, complex cases; however, current data are derived primarily from case reports and small study groups, and do not justify presenting GBR as the standard procedure for every case of insufficient bone volume.

### 7.5. Closing OAC with Donor Third Molar

Once the recipient site has been adequately prepared and the donor tooth atraumatically extracted, the third molar is carefully positioned in the recipient socket. The tooth should be inserted with minimal manipulation, avoiding repeated in-and-out movements that may damage the periodontal ligament cells. Correct three-dimensional positioning must be verified both clinically and radiographically, ensuring adequate depth, axial inclination, and the absence of occlusal interference. Following placement, the transplanted tooth is stabilized using crossed mattress sutures over the occlusal surface; additional splinting may be applied if primary stability is insufficient. Splints can be removed after 2 to 6 weeks, depending on the clinical parameters (periotest values, percussion sound) [42].

In cases of completed root development, endodontic treatment is recommended within 7 to 14 days following transplantation, prior to pulp necrosis and associated periapical inflammation. Root canal treatment may also be performed immediately after extraction of the donor tooth and before placement into the recipient socket, although this approach is generally not advised due to prolonged extraoral time and its detrimental effect on periodontal ligament cell viability.

In cases of incomplete root development, endodontic intervention should be withheld, and pulp vitality monitoring initiated approximately 6 weeks post-procedure, as spontaneous revascularization remains possible.

### 7.6. Postoperative Care and Follow-Up Clinical Examinations

Detailed postoperative instructions should be provided to the patient both verbally and in written form. The following recommendations apply:

- Oral hygiene and wound care:

Patients should be advised to use a soft-bristled toothbrush in the area of the transplanted tooth, while avoiding excessive pressure on the surgical site. In cases where the transplanted tooth requires additional stabilization via splinting, meticulous plaque control must be maintained in the area of the splint and the transplanted tooth throughout the entire period the splint remains in the oral cavity—that is, for 2 to 6 weeks [42]. Chlorhexidine digluconate mouthwash (0.12–0.2%) is recommended for a short postoperative period, as it reduces bacterial load and supports wound healing [20,43]. Nevertheless, due to the potential for adverse effects, the duration of using chlorhexidine products should be limited and based on individual clinical assessment. Gentle saline rinses may additionally be introduced.

- Diet:

A soft or semi-liquid diet is recommended for a minimum of 2–3 weeks following the procedure. Hard, crunchy, and sticky foods should be avoided to prevent mechanical destabilization of the transplanted tooth. Hot beverages and alcohol should be withheld during the initial healing period.

- Pharmacotherapy:

Postoperative antibiotic therapy should not be administered routinely but rather based on clinical indications. Given that the procedure in question involves opening the maxillary sinus lumen, single-dose antibiotic prophylaxis (2000 mg of amoxicillin orally 30–60 min before the procedure, the so-called ‘one shot’ regimen, or 600 mg of clindamycin for patients allergic to penicillin-class antibiotics) is explicitly recommended for this indication by the PTS/NPOA guidelines on alveolar process surgery. A multi-day course of antibiotics (3–7 days) is justified only in cases presenting with systemic symptoms of odontogenic infection, i.e., fever, malaise, tendency for the infection to spread, as well as in the case of immunocompromised patients, in accordance with the PTS/NPOA guidelines on the treatment of odontogenic infections. In cases requiring therapeutic antibiotic treatment, the choice of drug should be based on the clinical state, the nature of the infection, the type of reported penicillin allergy, local resistance patterns, and current guidelines for treating odontogenic infections. A detailed distinction between SSI prophylaxis, IE prophylaxis, and the therapeutic use of antibiotics is presented in Table 1. Analgesic and anti-inflammatory therapy with ibuprofen (400–600 mg every 6–8 h, as needed) is recommended for postoperative pain management. Nasal decongestant drops (e.g., xylometazoline) may be prescribed for 5–7 days to reduce sinus mucosal edema and facilitate drainage.

- Behavioral recommendations:

Patients should avoid nose blowing and sneezing with their mouths closed. If sneezing is unavoidable, it should be performed with the mouth open and without occluding the nostrils, as increased intraoral or intranasal pressure may displace the transplanted tooth or disrupt the OAC closure. Smoking and the use of drinking straws are also not recommended.

Radiological follow-up should be performed according to a structured protocol. An initial periapical or panoramic radiograph should be taken immediately post-operatively to assess the position of the transplanted tooth and establish a baseline for the surrounding bony structures and the maxillary sinus floor. A follow-up radiograph at 6 weeks is recommended, coinciding with pulp vitality assessment in cases of teeth with incomplete root development [27,28]. At 3 months, the early periodontal response should be evaluated, with attention to the presence of the periodontal ligament space and early signs of corticalisation at the bone-socket interface. The 6-month examination constitutes a critical checkpoint for detecting root resorption, assessing sinus floor continuity, and confirming the maintenance of normal periodontal space width. The 1-year follow-up, as applied by Assad et al. [20], serves as the primary endpoint for evaluating overall procedural success, including OAC closure and osseous integration. Given the risk of late inflammatory or replacement resorption, annual radiographic examinations are advised for a minimum of 4 to 5 years following the procedure, in accordance with the guidelines of Andreasen et al. [26]; long-term follow-up extending beyond this period may further confirm the stability of the outcome, as demonstrated by Grün et al., who reported sinus floor remodeling observed over 19 years post-transplantation [19]. In cases where standard radiographs fail to demonstrate the expected healing response, or where sinus pathology is suspected, CBCT imaging should be considered due to its superior sensitivity in assessing three-dimensional alveolar geometry and maxillary sinus mucosal status [41]. Follow-up visits should also include a mobility degree check-up using Periotest.

## 8. Discussion

The presented review indicates that third molar autotransplantation can serve as an alternative treatment approach in selected cases of OACs, particularly when a suitable donor tooth is available and anatomical conditions allow for its atraumatic extraction and stable placement at the recipient site. A distinctive feature of this method is the ability to simultaneously close the OAC and replace the missing tooth, distinguishing it from most conventional surgical techniques that focus primarily on closing the soft-tissue defect. Available clinical reports describe favorable outcomes, including OAC closure, survival of the transplanted tooth, and regeneration of bone structures at the maxillary sinus floor. While these data suggest the potential clinical value of autotransplantation, they should be interpreted with caution due to the limited number of reported cases and the absence of comparative studies.

The analysis of the available literature indicates that the key elements of the procedure are proper patient selection and the assessment of both the donor tooth and the recipient site. The donor tooth should possess an anatomy that allows for the least traumatic extraction possible, as preserving the integrity and viability of periodontal ligament cells is a fundamental factor for proper healing following autotransplantation. Adequate space at the recipient site, the absence of active inflammation, and the ability to achieve a stable tooth position without excessive bone preparation are also essential. Detailed preoperative radiographic assessment—including the use of CBCT—enables analysis of the donor tooth and recipient site anatomy and can facilitate the identification of potential difficulties prior to the procedure. Consequently, the proposed algorithm places particular emphasis on the patient selection phase before a decision is made to use autotransplantation to close OAC.

Another key aspect of the proposed approach is to minimize trauma to the donor tooth as well as the time it spends outside the socket. Repeatedly inserting the tooth into the recipient site to check the fit can cause mechanical damage to the periodontal ligament; therefore, site preparation should be as conservative as possible and, ideally, performed prior to extracting the donor tooth. In this context, digital planning and the use of a 3D-printed tooth replica can facilitate more precise socket preparation, reduce the number of trial fittings, and shorten the extra-alveolar time. However, as available evidence regarding the superiority of 3D technology remains limited, its use should not be considered a mandatory part of the procedure. Similarly, in cases of insufficient bone volume, the decision regarding additional regenerative procedures should be made on an individual basis, and guided bone regeneration (GBR) should not be presented as a routine measure for every instance of bone tissue deficiency.

The success of autotransplantation depends not only on the procedure itself but also on proper postoperative management. Once the tooth is placed in the recipient site, adequate stability must be ensured while avoiding excessive or prolonged rigid splinting and occlusal overloading. Plaque control is also a crucial element; the patient should maintain good oral hygiene and gently clean the surgical site—as well as the area around the splint, if one was used, throughout the period the splint remains in place. The use of antiseptic agents, including chlorhexidine, can supplement mechanical plaque control during the initial healing phase; however, the duration of use should be limited and tailored to the clinical situation. Long-term clinical and radiographic follow-up is essential due to the risk of late complications, particularly root resorption and ankylosis. The stage of donor tooth root development and the potential for pulp revascularization should be taken into account prior to performing endodontic treatment.

A key strength of this paper is its focus on the rarely described application of tooth autotransplantation—a procedure that has received relatively little attention in broader existing studies on the treatment of OACs. The paper compiles data regarding patient qualification and donor tooth selection, recipient site preparation, surgical techniques, endodontic treatment, postoperative care, and long-term follow-up. Based on this synthesis, a structured clinical algorithm is proposed; its aim is not only to facilitate decision-making in clinical scenarios but also to highlight procedural aspects where available evidence remains insufficient. An additional value of the work is the inclusion of modern technologies that help autotransplantation planning—such as CBCT imaging and the use of 3D-printed donor tooth models—while critically acknowledging the limited evidence supporting these approaches.

However, this review has significant limitations resulting primarily from the quality and number of available publications. The largest available clinical study, conducted by Assad et al., involved only 20 patients with a 12-month follow-up period, while the remaining data consist mainly of single case reports or small case series. Small study groups, the observational nature of the data, heterogeneity in surgical techniques and postoperative care, and the lack of comparative studies currently preclude definitive conclusions regarding the efficacy and predictability of this method. A significant limitation of the current evidence is the absence of a standardized antibiotic regimen specific to third-molar autotransplantation for OAC closure. The analyzed reports employed various perioperative and postoperative antibiotic regimens; however, their heterogeneity and the low level of evidence preclude recommendations regarding a specific agent or duration of treatment. Therefore, decisions concerning antibiotic use and selection should take into account the specific clinical indication, the nature of the infection, individual patient factors, local resistance patterns, and current guidelines, in accordance with the principles of rational antibiotic therapy.

Another limitation is the incomplete reporting of key clinical parameters, including OAC dimensions, donor tooth storage methods, recipient-site preparation, and details of postoperative care. These limitations also affect the present work, as the proposed algorithm was developed from heterogeneous, low-level evidence. Consequently, it should be regarded as an expert-based framework derived from a synthesis of OAC-specific data and broader knowledge of tooth autotransplantation and oral surgery, rather than as a validated standard of care. Larger prospective studies with standardized inclusion criteria, detailed reporting of surgical techniques and postoperative care, and long-term follow-up are needed to determine the efficacy, safety, and predictability of this approach and to validate the proposed algorithm.

In conclusion, autotransplantation of a third molar may serve as an alternative therapeutic option in selected cases of OAC; however, the current level of evidence does not allow its predictability to be reliably determined or evidence-based guidelines to be formulated. The proposed algorithm should therefore be regarded as an expert-based framework to support individual clinical decision-making and serve as a basis for further prospective validation. Further clinical research is essential to establish the role of this approach in OAC treatment.

## Figures and Tables

**Table 1:** Antibiotic use in third molar autotransplantation for OAC closure.

Clinical Indication	Patient Qualification	Recommended Regimen	Important Considerations	Source
SSI prophylaxis—opening of the maxillary sinus	Patients undergoing surgery involving the opening of the maxillary sinus lumen	Amoxicillin 2000 mg p.o., single dose 30–60 min before surgery. In penicillin allergy, PTS/NPOA lists cefazolin 1000 mg or clindamycin 600 mg.	Applies to SSI prophylaxis, not IE prophylaxis, and is not specific to tooth autotransplantation. Clindamycin should not be considered a universal alternative.	PTS/NPOA [30]
IE prophylaxis	Patients at high risk of IE undergoing at-risk dental procedures, including oral surgery.	Amoxicillin 2000 mg p.o., single dose 30–60 min before the procedure. In penicillin allergy: cephalexin 2000 mg, azithromycin/clarithromycin 500 mg, or doxycycline 100 mg; parenteral alternatives: cefazolin or ceftriaxone 1000 mg.	Clindamycin is not recommended. Cephalosporins should not be used after previous anaphylaxis, angioedema, or urticaria to penicillin/ampicillin.	ESC 2023 [34]
Routine postoperative antibiotic therapy	Patients without clinical signs of spreading or systemic infection.	Not routinely recommended. OAC/autotransplantation reports describe postoperative courses of 3–7 days, but evidence is insufficient to establish a procedure-specific regimen.	Regimens from case reports/small series should not be interpreted as evidence-based recommendations.	Clinical studies included in the present review
Therapeutic antibiotic treatment	Systemic involvement (e.g., fever, malaise), spreading odontogenic infection, or relevant immunocompromised status.	Antibiotic selection and duration should be individualized according to the clinical condition and current recommendations for odontogenic infections.	Consider infection type, nature of the penicillin allergy, local resistance patterns, and antimicrobial stewardship.	PTS/NPOA [30]; ESE [31]

**Table 2:** Advantages and limitations of the presented method.

Advantages	Limitations
Restoration of masticatory function	An atraumatic extraction is essential not to damage the donor tooth—in cases of complicated anatomy or impacted teeth, it can be problematic
In case of failed autotransplantation, many alternative methods are available	This procedure can be applied only in cases of minor OACs and those restricted to a single tooth: narrow therapeutic indications
Due to transplantation of one’s own tooth, the treatment is biocompatible, thus has a high chance of integration	Space limitations for the donor tooth among adjacent teeth
Similar rules for performing the procedure as in the case of surgical extraction	Third molar presence is essential
Cost-effective method compared to implants	Mucoperiosteal tissue must not be damaged
Bone preservation	Patient needs to maintain satisfactory oral hygiene, i.e., Approximal Plaque Index has to be lower than 15%
Potential for better aesthetics	

**Table 3:** Details and results of the mentioned studies.

Author	Patient (Age, Sex)	Procedure Decision and Timing	Donor Tooth Stabilization	Pharmacological Treatment	Root Canal Treatment	Further Treatment	Complications	Apical Formation of Donor Tooth
Nagori et al. [21]	22 F	Decision based on radiograph after OAC occurred	Splinted with composite and metal wire	Amoxicillin 3x/d, antihistamine and analgesic for 3 days	After 3 weeks	Crown after 6 months	None mentioned	Complete
Kitagawa et al. [9]	31 F	Immediately after the extraction and OAC confirmed	Firm finger pressure and light tapping with a mallet	Perioperative antibiotic—exact drug not mentioned	After 3 weeks	Prosthetic treatment after 5 months	After 3 years—none	Complete
Kitagawa et al. [9]	30 F	Autotransplantation as an alternative to implantation due to insufficient alveolar crest of the maxilla, decision before OAC occurred	Firm finger pressure and light tapping with a mallet	Perioperative antibiotic—exact drug not mentioned	After 3 weeks	Prosthetic treatment after 5 months	After 2 years—none	Complete
Lucidarme et al. [24]	16 M	Autotransplantation of 18 in 16 socket (tooth qualified for extraction due to ankylosis), decision before extraction	Suture stabilization + 4/10 steel bar	Perioperative antibiotic—exact drug not mentioned	None—the donor tooth maintained its vitality	Orthodontic treatment probably 2 months after	After 1 year—none except slight mesio-distal bone loss	Incomplete
Grün et al. [19]	20 F	Transplantation as an alternative to implantation due to insufficient alveolar crest of the maxilla, decision before OAC occurred	Sutures	0.12% chlorhexidine solution, antibiotic therapy, anti-inflammatory medications as needed	None	None mentioned	After 19 years—massive resorption	No confirmation—probably complete
Assad et al. [20]	20–40, M 65% and 35% F (20 patients)	Patients with extractions resulting in OACs were referred to the maxillofacial clinic in order to perform the treatment	Splinting wire + X-shaped suturing over the tooth	amoxicillin, ibuprofen, mouthwash (not specified)	After extraction, before autotransplantation	None mentioned	Failure in the case of 2 patients (10%)	Probably complete in most cases
Bumairemu et al. [40]	17–59, M 33.3%, F 66.6% (12 patients)	Immediate autotransplantation after OAC diagnosis	Stabilization with a figure-of-eight suture	Prophylactic antibiotics administered preoperatively; postoperative antibiotic therapy for 3–5 days; mouth rinse and hygiene instructions	Performed after 2–4 weeks, depending on tooth stability and vitality; the paper does not specify whether all autotransplanted teeth underwent RCT	None mentioned	After 12 months—none	No confirmation—probably completed

## Data Availability

No new data were created or analyzed in this study. Data sharing is not applicable to this article.

## Abbreviations

The following abbreviations are used in this manuscript:

3D	Three-dimensional
CBCT	Cone-beam computed tomography
CT	Computed tomography
EPT	Electric pulp testing
GBR	Guided bone regeneration
IE	Infective endocarditis
LDF	Laser Doppler flowmetry
MRONJ	Medication-related osteonecrosis of the jaw
OAC	Oroantral communication
OAF	Oroantral fistula
OPG	Orthopantomogram
PLA	Polylactic acid
PRF	Platelet-rich fibrin
RCT	Root canal treatment
SSI	Surgical site infection
